# The sodium channel subunit SCNN1B suppresses colorectal cancer via suppression of active c-Raf and MAPK signaling cascade

**DOI:** 10.1038/s41388-022-02576-4

**Published:** 2022-12-23

**Authors:** Yun Qian, Lianxin Zhou, Simson Tsz Yat Luk, Jiaying Xu, Weilin Li, Hongyan Gou, Huarong Chen, Wei Kang, Jun Yu, Chi Chun Wong

**Affiliations:** 1 Department of Gastroenterology and Hepatology, Shenzhen University General Hospital, Shenzhen, China; 2 Institute of Digestive Disease, Department of Medicine and Therapeutics, State Key Laboratory of Digestive Disease, Li Ka Shing Institute of Health Sciences, The Chinese University of Hong Kong, Hong Kong SAR, China; 3 Department of Anatomical and Cellular Pathology, The Chinese University of Hong Kong, Hong Kong SAR, China

**Keywords:** Colorectal cancer, Cancer genetics, Growth factor signalling

## Abstract

The incidence of colorectal cancer (CRC) is rising worldwide. Here, we identified SCNN1B as an outlier down-regulated in CRC and it functions as a tumor suppressor. SCNN1B mRNA and protein expression were down-regulated in primary CRC and CRC cells. In a tissue microarray cohort (*N* = 153), SCNN1B protein was an independent prognostic factor for favorable outcomes in CRC. Ectopic expression of SCNN1B in CRC cell lines suppressed cell proliferation, induced apoptosis, and cell cycle arrest, and suppressed cell migration in vitro. Xenograft models validated tumor suppressive function of SCNN1B in vivo. Mechanistically, Gene Set Enrichment Analysis (GSEA) showed that SCNN1B correlates with KRAS signaling. Consistently, MAPK qPCR and kinase arrays revealed that SCNN1B suppressed MAPK signaling. In particular, SCNN1B overexpression suppressed p-MEK/p-ERK expression and SRE-mediated transcription activities, confirming blockade of Ras-Raf-MEK-ERK cascade. Mechanistically, SCNN1B did not affect KRAS activation, instead impairing activation of c-Raf by inducing its inhibitory phosphorylation and targeting active c-Raf for degradation. The ectopic expression of c-Raf fully rescued cell proliferation and colony formation in SCNN1B-overexpressing CRC cells, confirming c-Raf as the principal molecular target of SCNN1B. In summary, we identified SCNN1B as a tumor suppressor by functioning as a c-Raf antagonist, which in turn suppressed oncogenic MEK-ERK signaling.

## Introduction

Colorectal cancer (CRC) is third most common cancer globally [1] and CRC is a devastating disease if diagnosed at an advanced stage. Moreover, CRC is increasingly diagnosed in younger adults [2], highlight an urgent need to improve our understanding of the pathogenesis of this disease. Genetic and epigenetic aberrations have been shown to play a role in colorectal tumorigenesis [3], but the key genes involved in CRC pathogenesis are far from complete. To unravel novel tumor suppressor genes involved in CRC, we analyzed the outlier genes down-regulated in human CRC tissues compared to adjacent normal colon tissues, revealing that SCNN1B as an outlier significantly silenced in CRC. We further identified that SCNN1B is a novel gene whose promoter is densely methylated in human CRC. Nevertheless, its potential role in CRC remains largely unknown.

SCNN1B is located on chromosome 16p12.2 and it encodes β-subunit of the epithelial sodium channel (ENaC). Our previous work has shown that SCNN1B suppresses gastric cancer [4], but the functional importance and molecular mechanism of SCNN1B in CRC is largely unclear. Here, we demonstrated that SCNN1B was consistent down-regulated in CRC using multiple patient cohorts, concomitant with its promoter hypermethylation. Moreover, SCNN1B protein expression and DNA methylation status are independent prognostic factor that predicts survival of CRC patients. Using a series of in vitro and in vivo studies, we found that SCNN1B exerted a tumor suppressor function by suppressing cell proliferation by inducing cell cycle arrest and apoptosis. Mechanistically, we revealed that SCNN1B (i) promoted the proteasome-mediated degradation of active c-Raf, a key upstream regulator of oncogenic MAPK signaling pathway, which in turn, (ii) suppressed MEK-ERK and AKT cascade; and (iii) the downstream effects leading to cell cycle arrest and apoptosis. Finally, we showed that SCNN1B overexpression improves sensitivity of CRC cells to chemotherapy. Taken together, our work has identified SCNN1B as a novel tumor suppressor in CRC via the repression of c-Raf mediated MEK-ERK signaling cascades and is a therapeutic target.

## Results

### SCNN1B is downregulated in CRC and is an independent predictor of favorable outcome in CRC patients

**Fig. 1:**
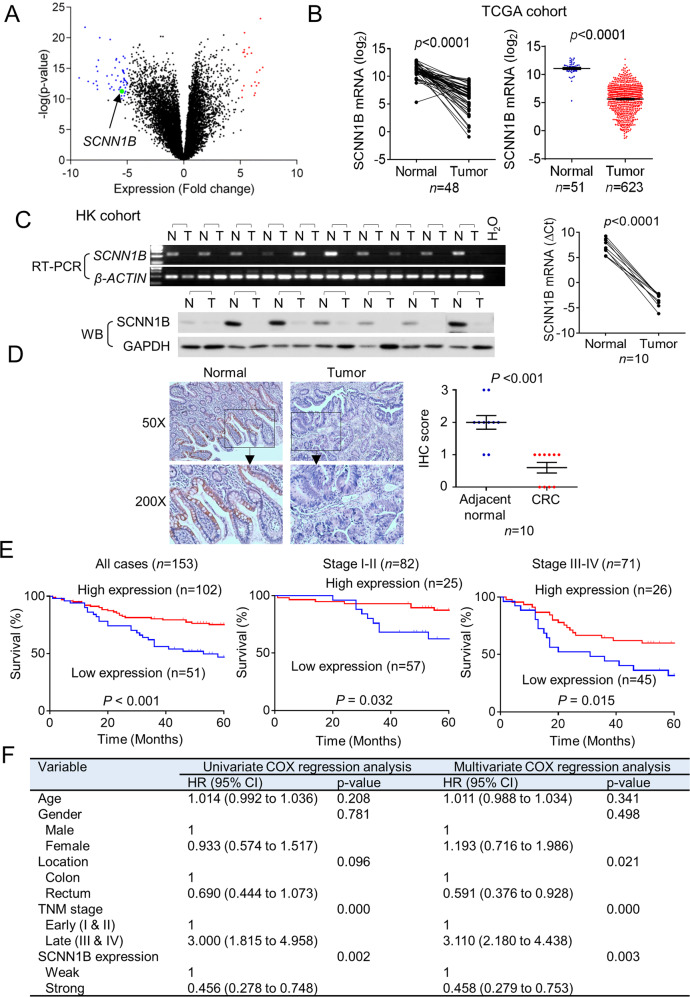
SCNN1B is silenced in CRC and is associated with patient survival. **A** Volcano plot of RNA-sequencing dataset from the Cancer Genome Atlas (TCGA) CRC cohort, showing that SCNN1B is an outlier gene down-regulated in CRC. **B** SCNN1B mRNA is up-regulated in TCGA CRC dataset in both paired samples (*P* < 0.0001) and the overall cohort (*P* < 0.0001). **C** RT-PCR and western blot validated that SCNN1B mRNA and protein are silenced in CRC tissues compared to adjacent normal colon tissues (left). qPCR validation in paired CRC and adjacent normal samples (right). **D** Immunohistochemistry staining showed that SCNN1B is down-regulated in CRC compared to adjacent normal samples. **E** Tissue microarray (TMA) CRC cohort for SCNN1B protein expression. Representative Kaplan-Meier survival plots of SCNN1B expression in CRC revealed that high SCNN1B expression is correlated with improved survival in the overall cohort (left), early-stage (middle), and late-stage CRC (right). **F** Cox-regression including univariate and multivariate analysis demonstrated that SCNN1B is an independent predictor of favorable survival in CRC patients.

To uncover potential tumor suppressor genes in CRC, we analyzed RNA-seq dataset from the Cancer Genomic Atlas (TCGA), revealing that SCNN1B is an outlier gene down-regulated in CRC compared to adjacent normal tissues (fold change < −5; *P* < 1 × 10^−10^) (Fig. 1A). SCNN1B is downregulated in both paired CRC and adjacent normal tissues (*N* = 48, *P* < 0.0001) and the overall CRC cohort (*P* < 0.0001) (Fig. 1B). We next validated these results in our local cohort. RT-PCR and qPCR analyses of 10 paired CRC and adjacent normal tissues verified significant downregulation of SCNN1B mRNA in CRC (Fig. 1C). We next sought to confirmed down-regulated SCNN1B at protein level. Western blot (*N* = 7, *P* < 0.001) and immunohistochemistry (*N* = 10, *P* < 0.001) confirmed that SCNN1B is significantly downregulated in CRC compared to their corresponding adjacent normal tissues (Fig. 1D). Besides CRC, SCNN1B was down-regulated in multiple cancer types in TCGA cohort, implying a general tumor suppressive role of this protein (Figure S1).

To investigate the clinical significance of SCNN1B in CRC, we next assessed the association of SCNN1B protein expression with clinicopathologic features and patient outcomes in a tissue microarray cohort (TMA) of CRC (*N* = 153) [5, 6]. Kaplan-Meier curve analysis demonstrated that high cytoplasmic SCNN1B is associated with better overall survival in CRC (*P* < 0.001) (Fig. 1E). Further stratification of TMA cohort into early stage (TNM stage I/II) and late stage (TNM stage III/IV) revealed that high SCNN1B protein expression is associated with better survival in early (*P* < 0.05) and late-stage (P < 0.05) CRC (Fig. 1E). We next performed univariate and multivariate Cox regression analysis (Fig. 1F). In univariate Cox regression, high SCNN1B protein expression was associated with better overall survival in CRC [HR: 0.456; 95% confidence interval (C.I.): 0.278–0.748, *P* = 0.002]. Apart from SCNN1B protein expression, tumor stage (*P* < 0.001) was correlated with worse survival by univariate analysis. After adjustment for potential confounding factors including age, gender, tumor location, and TNM stage, SCNN1B protein expression was found to be an independent prognostic factor for improved survival by multivariate Cox regression analysis [HR: 0.458; 95% C.I.: 0.279–0.753, *P* = 0.003]. Taken together, a high SCNN1B protein expression predicts favorable prognosis in patients with CRC

### SCNN1B is silenced by promoter methylation in CRC and its methylation status serves as an independent predictor of poor survival

**Fig. 2:**
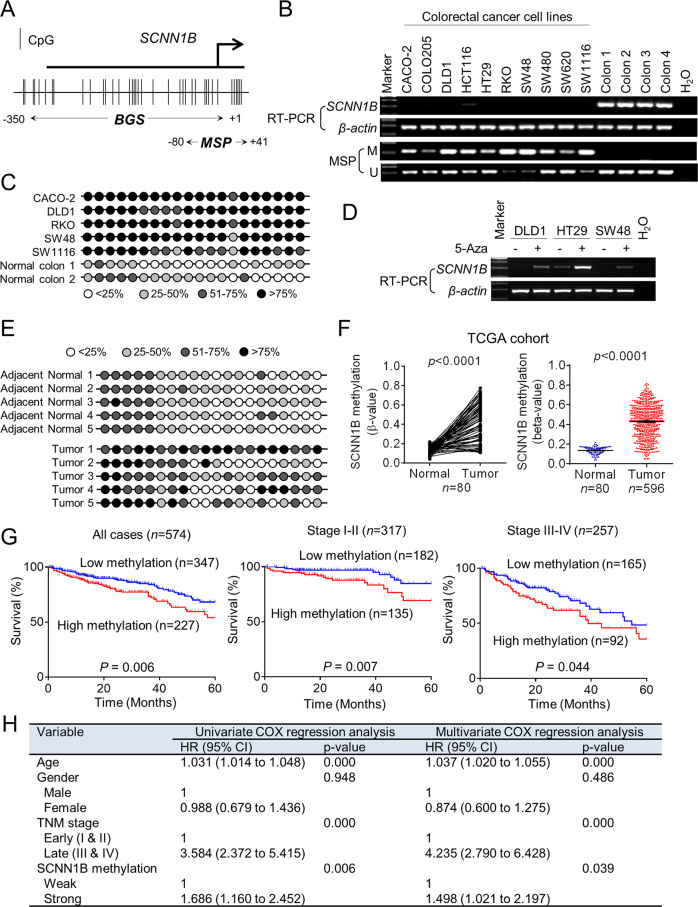
SCNN1B promoter hypermethylation mediates its transcriptional silence and is associated with patient survival. **A** The design of bisulfite genomic sequencing (BGS) and methylation-specific PCR (MSP) for SCNN1B promoter region. **B** RT-PCR and MSP of 10 CRC cell lines and 4 normal colon tissues. SCNN1B mRNA is silenced in CRC, concomitant with marked promoter hypermethylation. **C** DNA demethylation reagent, 5-Aza, restored SCNN1B mRNA in CRC cell lines. **D** BGS confirmed SCNN1B promoter hypermethylation in CRC cells as compared to normal colon. SCNN1B is hypermethylated in CRC tumor tissues as compared to paired adjacent normal colon tissues. **E** Analysis of TCGA CRC cohort (DNA methylation) showed that SCNN1B is hypermethylated in paired samples (left) and the overall cohort (right). **F** Integration of RNA-seq and DNA methylation in TCGA cohort revealed that SCNN1B expression is negatively correlated with its promoter methylation. **G** Kaplan-Meier curve analysis showed that high SCNN1B promoter hyper-methylation predicts poor survival of CRC patients (left), including early-stage (middle) and late-stage (right) CRC. **H** Univariate and multivariate Cox regression analysis validated SCNN1B as an independent prognostics factor for poor survival in CRC.

Our previous work has shown that SCNN1B is readily expressed in normal tissues with strong expression in the colon [4]. Given that promoter methylation is a prominent factor in silencing of tumor suppressor genes, we next asked if SCNN1B promoter was hypermethylated in CRC. We thus performed bisulfite genomic sequencing (BGS) and methylation-specific PCR (MSP) analysis of the SCNN1B promoter (Fig. 2A). As shown in Fig. 2B, SCNN1B mRNA is silenced in all CRC cell lines investigated (10 out of 10), whereas its expression can be readily detected in normal colon samples (*N* = 4). MSP demonstrated dense methylation of SCNN1B promoter methylation in CRC cell lines, whilst normal colon tissues were largely unmethylated (Fig. 2B). BGS of 20 CpG sites in the SCNN1B promoter showed very high levels (>75%) of promoter methylation in CRC cell lines, in contrast to lower promoter methylation in normal colon tissues (Fig. 2B). To validate that the hypermethylation of SCNN1B promoter directly contributes to transcriptional silence of SCNN1B, we treated a panel of 3 CRC cell lines with 5-Aza-2′-deoxycytidine (5-Aza), a DNA methyltransferase (DNMT) inhibitor. Consistent with our hypothesis, 5-Aza significantly restored SCNN1B mRNA expression in all CRC cell lines (Fig. 2C), implying that SCNN1B promoter hypermethylation contributes to its silencing in CRC. Combination of 5-Aza plus trichostatin A (TSA), a histone deacetylase inhibitor, further restored SCNN1B mRNA expression (Figure S2), suggesting that DNA methylation in concert with histone deacetylation to silence SCNN1B. We next examined SCNN1B methylation status in primary CRC. BGS demonstrated that SCNN1B promoter methylation was significantly induced in CRC compared to paired adjacent normal colon tissues (Fig. 2D). Analysis of TCGA CRC DNA methylation datasets revealed highly consistent hypermethylation of SCNN1B promoter in paired CRC and adjacent normal tissues (*N* = 80, *P* < 0.0001), as well as in the overall cohort (*P* < 0.0001) (Fig. 2E). Finally, the analysis of TCGA CRC dataset revealed an inverse correlation between SCNN1B mRNA expression and promoter methylation (R = −0.5106, *P* < 0.0001) (Fig. 2F), suggesting that promoter methylation is associated with SCNN1B silencing in humans.

Prognostic value of SCNN1B promoter methylation status was next determined in the TCGA CRC cohort. As shown in Fig. 2G, SCNN1B high promoter methylation predicts the poor survival of CRC patients (*N* = 574, *P* = 0.006) by Kaplan-Meier curve. Stratification of the patient cohort into early (stage I/II) and late (stage III/IV) showed that SCNN1B promoter methylation is associated with poor survival in early (*P* < 0.007) and late CRC (*P* < 0.044) (Fig. 2G). We next performed univariate and multivariate Cox regression analysis (Fig. 2H). In univariate Cox regression analysis, a high SCNN1B promoter methylation was associated with worse overall survival in CRC [HR: 1.686]; 95% C.I.: 1.160–2.452 *P* = 0.006. Tumor stage (*P* < 0.001) and age (*P* < 0.001) were also correlated with survival by univariate analysis. After adjustment for potential confounding factors, including age, gender, and TNM stage, SCNN1B promoter methylation was found to be an independent prognostic factor for poor survival by multivariate Cox regression analysis [HR: 1.498; 95% C.I.: 1.021–2.197, *P* = 0.039]. Hence, high SCNN1B promoter methylation predicts poor prognosis in patients with CRC

### SCNN1B functions as a tumor suppressor in CRC cell lines

**Fig. 3:**
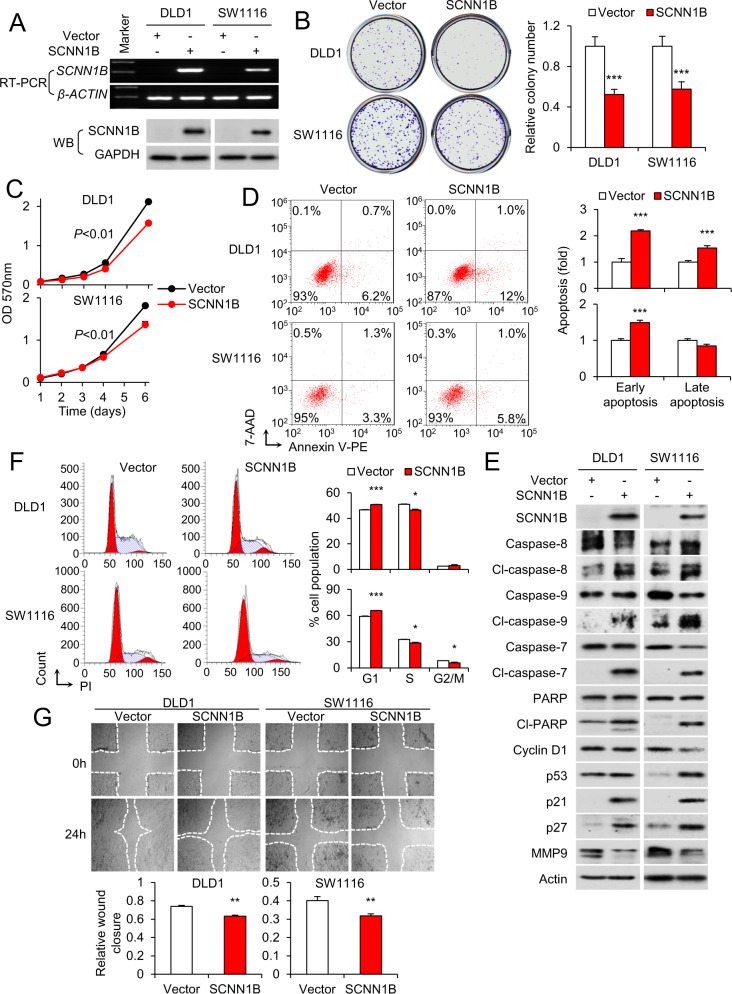
SCNN1B functions as a tumor suppressor in CRC cells via a cytokinetic effect. **A** Overexpression of SCNN1B in DLD1 and SW1116 cells. **B** SCNN1B overexpression suppressed colony formation and **C** cell growth in DLD1 and SW1116 cells. **D** SCNN1B ectopic expression induced both early and late apoptosis in DLD1 cells, and promoted early apoptosis in SW1116 cells. **E** Western blot of apoptosis and cell cycle markers. SCNN1B increased cleaved caspase-8, caspase-9, caspase-7, and PARP indicative of apoptosis. SCNN1B suppressed Cyclin D1, whilst inducing p53, p21 and p27. SCNN1B inhibited MMP9, a marker for cell migration. **F** SCNN1B promoted G1-S cell cycle arrest in DLD1 and SW1116 cells, as evidenced by increased cells in G1 phase and decreased proportion of cells in S phase. **G** SCNN1B overexpression suppressed cell migration, as determined by wound healing assay. Values represent means ± S.E.M. *P < 0.05, **P < 0.01, ***P < 0.0001.

The transcriptional silencing of SCNN1B in CRC, together with its consistent associations with patient survival lend us to hypothesize that SCNN1B may function as a novel tumor suppressor in CRC. To investigate this, we generated SCNN1B stably expressing DLD1 and SW1116 cell lines. Ectopic expression of SCNN1B was validated by qPCR and western blot (Fig. 3A). SCNN1B overexpression was found suppress colony formation (Fig. 3B) and cell viability by cell growth curve analysis (Fig. 3C) in both DLD1 and SW1116 cell lines. To understand biological basis of SCNN1B in suppressing CRC cell growth, we analyzed apoptosis and cell cycle distribution by flow cytometry. In DLD1 and SW1116 cells, overexpression of SCNN1B significantly induced apoptosis, as determined by Annexin V-7-AAD staining (Fig. 3D). In particular, SCNN1B strongly induced early apoptosis in both cell lines (*P* < 0.0001). Consistent with this, western blot showed that SCNN1B overexpression induced the expression of cleaved forms of caspase-8, caspase-9, caspase-7, and PARP (Fig. 3E), consistent with concomitant of intrinsic and extrinsic apoptosis pathway by SCNN1B. Cell cycle analysis demonstrated the accumulation of CRC cells in G1 phase following SCNN1B overexpression (*P* < 0.0001), with corresponding reduction in S phase population (Fig. 3F), suggesting G1 cell cycle arrest. In agreement with this, western blot showed that cell cycle checkpoints p21, p27, and p53 were all upregulated in SCNN1B-overexpressing cells, whereas the expression of cyclin D1 was reduced (Fig. 3E). We also examined the effect of SCNN1B on cell migration using the wound healing assay. As shown in Fig. 3G, SCNN1B overexpression impaired wound closure in DLD1 and SW1116 cells. Although SCNN1B forms a part of the sodium channel ENaC (Figure S3), we found that SCNN1B overexpression had no consistent effect on other ENaC subunits (Figure S3), nor on the sodium content of CRC cells (Figure S4), suggesting that the tumor suppressor function of SCNN1B is independent of its role in ENaC. Collectively, these findings indicate SCNN1B as a functional tumor suppressor in CRC.

### SCNN1B negatively regulates MAPK signaling in CRC

**Fig. 4:**
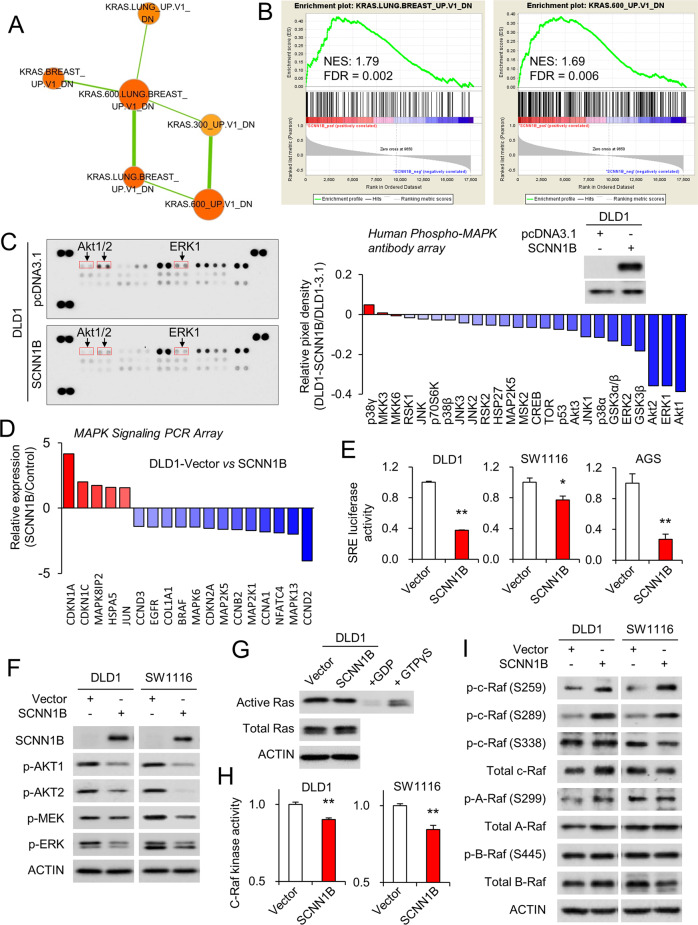
SCNN1B suppressed MAPK signaling via c-Raf-MEK-ERK cascade. **A** Gene set enrichment analysis (GSEA) with Cytoscape enrichmentMap revealed SCNN1B expression closely correlates with oncogenic KRAS signature in TCGA CRC RNA-seq dataset. **B** Enrichment plots showing that SCNN1B expression enriched for genes down-regulated by oncogenic KRAS. **C** MAPK antibody array analysis of DLD1 cells transfected with empty vector or SCNN1B demonstrated that SCNN1B down-regulated MAPK signaling, especially p-ERK1/2 and p-AKT. **D** MAPK signaling PCR array analysis of DLD1-vector and DLD1-SCNN1B cells revealed a consistent down-regulation of MAPK downstream genes, including cyclins and kinases. **E** DLD1, SW1116, and AGS cells expressing SCNN1B exhibited down-regulated SRE luciferase activity, an indicator of MAPK signaling. **F** Western blot validated that the overexpression of SCNN1B suppressed the phosphorylation (active) of AKT1, AKT2, MEK, and ERK, in agreement with the antibody and PCR data results. **G** Active KRAS pull-down assay revealed that SCNN1B had no effect on active KRAS activity. **H** c-Raf kinase activity was suppressed by SCNN1B overexpression in DLD1 and SW1116 cells. **I** Western blot of c-Raf, A-Raf, B-Raf, and their respective phosphorylated forms. SCNN1B induced the phosphorylation of c-Raf at S259 and S289, both of which are inactivating phosphorylation. It also reduced c-Raf S338 (active) phosphorylation. Besides, SCNN1B had no consistent effect on B-Raf or A-Raf expression and phosphorylation. Values represent means ± S.E.M.*P < 0.05, **P < 0.01, ***P < 0.0001.

To decipher the molecular mechanism of SCNN1B in CRC, we performed gene set enrichment analysis (GSEA) based on TCGA CRC cohort with SCNN1B as the marker gene. Among the oncogenic signature gene sets, we observed that multiple gene sets associated with oncogenic KRAS signature were enriched by SCNN1B, and they form a network by Cytoscape analysis (Fig. 4A). SCNN1B expression was most closely associated with downregulated genes upon KRAS activation in cancer cell lines (FDR < 0.01) (Fig. 4B). Given that KRAS is a central component of MAPK signaling pathway, we next performed Human Phospho-MAPK antibody array, which profiled the phosphorylation status of 24 MAPK kinases. By comparing DLD1-vector and DLD1-SCNN1B cells, we revealed that phosphorylation status of the vast majority of MAPK kinases were strongly downregulated by overexpression of SCNN1B (Fig. 4C). Densitometry analysis demonstrated that AKT1/2, ERK1/2, and GSK3β were downregulated by SCNN1B overexpression in DLD1 cells. To confirm that SCNN1B modulates MAPK signaling pathways, we next performed MAPK signaling PCR array (Fig. 4D). Concordantly, most of outlier genes were downregulated by SCNN1B, including cyclins (CCND2, CCNA1, CCND3) and MAPKs (MAP2K1/MEK1, MAP2K5/MEK5, MAPK13/p38, and MAPK6/ERK3) (Fig. 4D). To validate the effect of SCNN1B on the functional activity of MAPK signaling, we performed the serum-response factor (SRE) luciferase reporter assays. In DLD1, HCT116, and AGS cells harboring mutant KRAS, overexpression of SCNN1B all significantly inhibited SRE luciferase activities (Fig. 4E). In agreement with this, western blot confirmed that the expression of p-AKT1, p-AKT2, p-MEK1/2, and p-ERK1/2 (Fig. 4F). Taken together, these profiling assays imply that MAPK signaling is the downstream target of SCNN1B.

### SCNN1B mediates its tumor suppressive effect by suppressing c-Raf activation in CRC

In order to identify the specific signaling molecules modulated by SCNN1B, we first determine KRAS activation, a frequent cause of p-ERK activation in CRC. As shown in Fig. 4G, active KRAS pulldown assays revealed that SCNN1B had no significant effect on active KRAS levels in DLD1 cells. Evaluation of c-Raf kinase activity, downstream target of KRAS, revealed that SCNN1B suppressed c-Raf kinase activity (Fig. 4H). We hence analyzed expression and phosphorylation status of A-Raf, B-Raf, and c-Raf, the direct downstream molecules of KRAS, by Western blot (Fig. 4I). SCNN1B overexpression had no consistent effect on A-Raf, B-Raf, p-A-Raf, or p-B-Raf (Fig. 4I). On the other hand, SCNN1B consistently increased the phosphorylation of c-Raf at S259 and S289; whilst decreasing the phosphorylation at S338 (Fig. 4I). Re-expression of SCNN1B by demethylation reagent 5-Aza consistently restored S259 phosphorylation and inhibited S338 phosphorylation (Figure S5). Conversely, SCNN1B silencing in HCT116 cells reduced phospho-c-Raf (S259A) and induced phospho-ERK (Figure S6). Of particular interest, phosphorylation at S259 and S289 are feedback phosphorylation associated with inactivation of c-Raf; [7, 8] whilst S338 phosphorylation is a well-established activation site [8]. However, c-Raf upstream kinase (Protein kinase A, PKA) and phosphatase (Protein phosphatase 2A) involved in regulating S259/S289 phosphorylation were not altered by SCNN1B (Figure S7). These results imply that SCNN1B might repress c-Raf activation in CRC, and such an effect are independent of its upstream kinase and phosphatase.

**Fig. 5:**
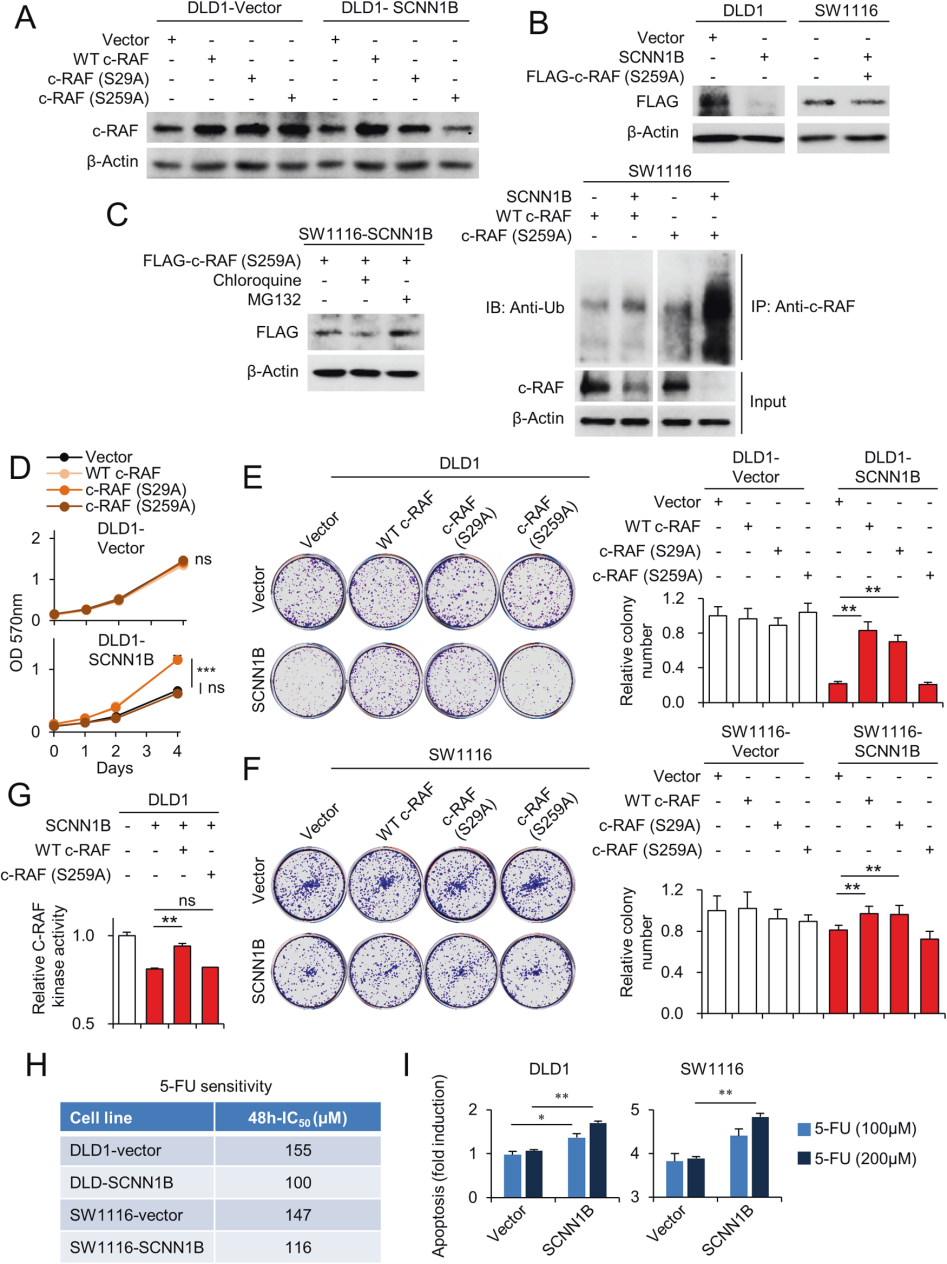
Re-expression of c-Raf reversed tumor suppressive effect of SCNN1B. **A** The re-expression of wildtype c-Raf and mutant c-Raf (S29A, S259A) in DLD1-vector and DLD1-SCNN1B cells. **B** SCNN1B overexpression suppressed the protein expression of S259A mutant c-Raf. **C** Addition of MG132, but not chloroquine, rescued c-Raf S259A protein expression in SCNN1B-expressing DLD1 cells (left). SCNN1B increased the ubiquitination of c-Raf S259A, but had no effect of wildtype c-Raf (right). **D** Ectopic expression of wildtype or mutant c-Raf had no effects on growth of DLD1-vector cells. For SCNN1B-overexpressing cells, wildtype or S29A c-Raf rescued cell proliferation. **E** In DLD1 and **F** SW1116 cells, overexpression of wildtype or S29A c-Raf reversed the inhibitory effect of SCNN1B on colony formation, but c-Raf S259A had no effect. **G** Re-expression of wildtype c-Raf, but not c-Raf S259A, rescued c-Raf kinase activity in SCNN1B-overexpressing DLD1 cells. **H** SCNN1B overexpression increased 48h-IC50 values of 5-Fluorouracil. **I** SCNN1B overexpression promoted apoptosis induction by 5-Fluorouracil. Values represent means ± S.E.M.*P < 0.05, **P < 0.01, ***P < 0.0001.

To functionally confirm that c-Raf as a target of SCNN1B, we performed rescue assays by the overexpression of wildtype c-Raf, c-Raf (S29A), or c-Raf (S259A), where S29A and S259A mutant abolished intrinsic negative feedback sites on c-Raf. Western blot validated successful overexpression of wildtype, S29A, and S259A c-Raf in wildtype cells (Fig. 5A). However, it was noted that the overexpression of wildtype or S29A c-Raf, but not S259A c-Raf, induced total c-Raf levels in SCNN1B-overexpressing cells (Fig. 5A). We next transfected FLAG-tagged c-Raf S259A into DLD1 and SW1116 control or SCNN1B-overexpressing cells and observed significant impaired c-Raf S259A expression only in cells overexpressing SCNN1B (Fig. 5B), despite successful transfection as determined by qPCR assay. This suggests that SCNN1B selectively suppressed the protein expression of constitutively active c-Raf S259A.

In addition, the supplementation of MG132, but not chloroquine, restored protein expression of c-Raf S259A in SCNN1B-overexpressing cells (Fig. 5C). In addition, SCNN1B increased the ubiquitination of c-Raf S259A, but not that of wildtype c-Raf (Fig. 5C), implying that SCNN1B might promote degradation of constitutively active c-Raf via ubiquitin-proteasome pathway. We next performed functional rescue assay in DLD1 and SW1116 cells. Cell viability assays showed that the ectopic expression of wildtype, S29A, or S259A c-Raf had no effect on DLD1 control cells; whilst either wildtype or S29A c-Raf, but not S259A c-Raf, rescued cell growth in SCNN1B-overexpressing cells (Fig. 5D). Colony formation assays in DLD1 and SW1116 cells demonstrated a consistent phenomenon. Overexpression of wildtype or S29A c-Raf, but not S259A c-Raf, largely restored colony formation capacity in SCNN1B-expressing DLD1 and SW1116 cell lines (Fig. 5E, F). Finally, we measured c-Raf kinase activity in DLD1 cells. As shown in Fig. 5G, ectopic expression of wildtype c-Raf restored c-Raf kinase activity in DLD1 cells. However, the overexpression of c-Raf S259A had no significant effect. Collectively, this indicates that SCNN1B can selectively target the activated c-Raf S259A for degradation, thereby suppressing its proliferative effect in KRAS-mutant CRC cells.

### SCNN1B promotes growth inhibition and apoptosis in combination with 5-Fluorouracil

Given that the activation of c-Raf/MEK/ERK signaling is associated with chemo-resistance [9, 10], we determined the effect of SCNN1B on the growth inhibitory effect of 5-FU. Consistent with our hypothesis, ectopic expression of SCNN1B in DLD1 and SW1116 cells reduced the 48h-IC_50_ values of 5-Fluorouracil (Fig. 5H). Moreover, we performed apoptosis assays in control and SCNN1B-expressing cells treated with or without 5-Fluorouracil (Fig. 5I). The overexpression of SCNN1B significantly suppressed the apoptosis of DLD1 and SW1116 cells treated with 5-Fluorouracil. Meanwhile, SCNN1B-overexpressing cells were less sensitive to ERK inhibitor U0126, a likely consequence of the already repressed MEK-ERK cascade when SCNN1B is overexpressed (Figure S8). SCNN1B had no effect on Vemurafenib, an inhibitor of BRAF, consistent with a lack of effect of SCNN1B on BRAF status (Figure S8). These data suggest that SCNN1B expression might promote drug response to chemotherapy in CRC.

### SCNN1B suppressed the tumorigenicity of CRC cells in vivo

**Fig. 6:**
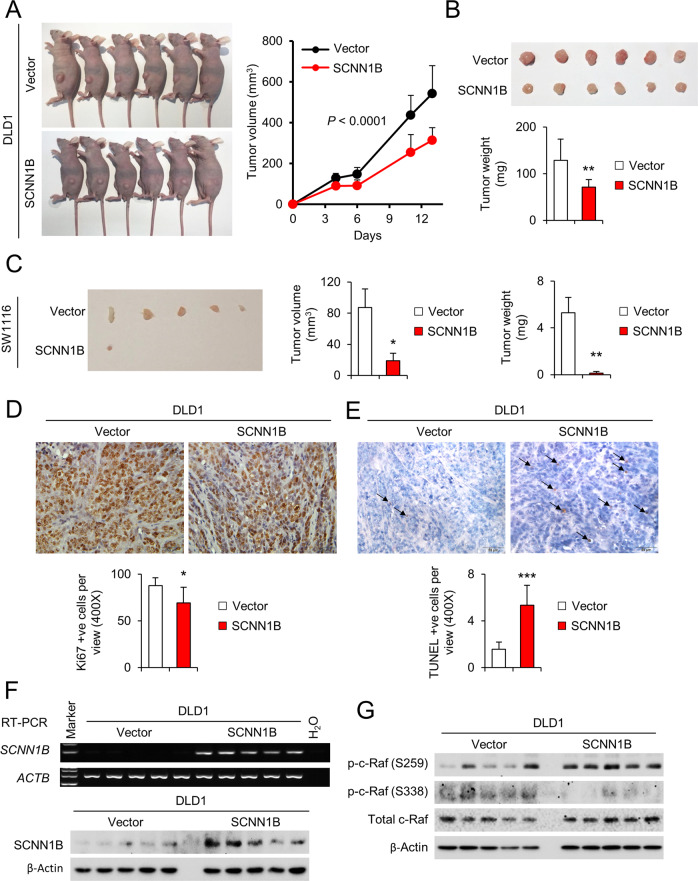
SCNN1B suppressed CRC growth in vivo. **A** DLD1-vector and DLD1-SCNN1B cells were subcutaneously implanted into nude mice. Representative images and tumor growth curve indicated that SCNN1B impaired the growth of tumor xenografts in vivo. **B** Images of tumors at the end point. Tumor weight was significantly reduced in SCNN1B overexpression group. **C** SCNN1B overexpression suppressed growth of SW1116 xenografts in nude mice. Representative images of tumors at the end point (left). The tumor volume (middle) and weight (right) were significantly reduced in SCNN1B overexpression group. **D** SCNN1B suppressed cell proliferation in DLD1 xenografts as determined by Ki-67 staining. **E** SCNN1B induced apoptosis in DLD1 xenografts as determined by TUNEL assay. **F** RT-PCR and Western blot demonstrated the successful overexpression of SCNN1B in tumor xenografts. **G** SCNN1B inactivated c-Raf in vivo, as indicated by increased S259 but reduced S338 phosphorylation. Values represent means ± S.E.M. *P < 0.05, **P < 0.01, ***P < 0.0001.

Given the promising in vitro results, we sought to investigate the effect of SCNN1B on CRC growth in vivo. We established SCNN1B stably expressing DLD1 and SW1116 cells, and then implanted empty vector or SCNN1B-expressing cells to the left and right flanks of nude mice, respectively. As shown in Fig. 6A, ectopic SCNN1B expression suppressed the growth of DLD1 xenografts (*P* < 0.0001). At the end point, tumor weight was also significantly suppressed in the SCNN1B-expressing tumors (*P* < 0.01) (Fig. 6B). Similarly, SCNN1B overexpression inhibited the growth of SW1116 xenografts, both in terms of tumor volume (*P* < 0.05) and tumor weight (*P* < 0.01) as compared to control tumors (Fig. 6C). Ki-67 staining and TUNEL assay demonstrated reduction of cell proliferation (*P* < 0.05) (Fig. 6D) and induction of apoptosis (*P* < 0.01) (Fig. 6E), respectively, in DLD1 tumors overexpressing SCNN1B. RT-PCR and Western blot confirmed the ectopic expression of SCNN1B in DLD1 xenografts (Fig. 6F). In addition, we examined c-Raf status by western blot. As shown in Fig. 6G, overexpression of SCNN1B in DLD1 xenografts induced p-c-Raf at S259, whereas phosphorylation at S338 was inhibited, implying that SCNN1B inactivated c-Raf activity in vivo, consistent with our in vitro findings.

## Discussion

In this study, we revealed that SCNN1B is an outlier gene that is silenced in human CRC as compared to normal colon tissues. A series of in vitro and in vivo studies demonstrated that SCNN1B exerts tumor-suppressive effects in CRC cell lines. Mechanistic investigations indicate that SCNN1B antagonizes c-RAF activation to abolish MEK-ERK and AKT signaling, the major oncogenic signaling cascades in CRC. These data illustrate the important role of SCNN1B in suppressing CRC development and its potential as a therapeutic target.

SCNN1B gene expression is absent in all the CRC cell lines examined, suggesting that SCNN1B might function as a tumor suppressor. In line with our hypothesis, overexpression of SCNN1B in CRC cell lines significantly impaired cell growth and colony formation in vitro. The growth of SCNN1B-expressing CRC cell xenografts was largely diminished, thus validating the tumor suppressive function of SCNN1B in vivo. We next investigated cytokinetic effects of SCNN1B in vitro. Ectopic expression of SCNN1B induced apoptosis, leading to cleavage of caspase-7 and PARP. Concomitantly, SCNN1B triggered G1-S cell cycle arrest, as evidenced by reduced cyclin D1 expression, together with increased expression of G1 checkpoints p21 and p27, which antagonize cyclin D1 [11]. The effect of SCNN1B on cytokinetics, therefore, underlies its tumor suppressive function in CRC.

We next elucidated the molecular mechanism of SCNN1B in CRC. GSEA pathway enrichment implies that SCNN1B correlates with MAPK signaling. Indeed, integrative PCR and antibody array analyses showed that SCNN1B negatively regulates activation of MEK-ERK and AKT signaling cascades. Given that MEK-ERK [12] is a well-established protumorigenic pathways that drives colorectal tumorigenesis, we next sought to identify the upstream elements targeted by SCNN1B. The classical ERK signaling pathways involved sequential activation of EGFR, RAS, and RAF proteins, which in turn amplify their signaling by phosphorylation of MEK and eventually ERK [13]. Whilst both DLD1 and SW1116 cells harbor mutant KRAS, SCNN1B had no effect on KRAS activation. On the other hand, our comprehensive evaluation of RAF proteins including A-Raf, B-Raf and C-Raf phosphorylation revealed that SCNN1B targets C-Raf in CRC cells. Specifically, SCNN1B overexpression tilted the balance of phosphorylation status of c-Raf from that of activation (S338) towards that of inhibition (S259 and S289), thus leading to dampened C-Raf kinase activity. Further rescue experiments with wildtype or mutant c-Raf lead to the discovery that SCNN1B targets constitutive active c-RAF (S259A, with the deletion of S259 phosphorylation site) for degradation, possibly via the ubiquitin-proteasome pathway. c-Raf is a proto-oncogene in cancer. Although it is infrequently mutated in CRC, the ablation of c-Raf has been shown to promote tumor regression in preclinical models of cancers involving MEK-ERK signaling activation [14–17]. Considering the importance of c-Raf for the survival of cancer cells in vitro and in vivo, numerous Raf inhibitors have been developed and successfully exploited in the clinic, such as Sorafenib [18] or Regorafenib [19], whilst selective c-Raf inhibitors is still undergoing preclinical and clinical evaluation [20, 21]. It would be of particular interest if SCNN1B expression might have an impact on efficacy of c-Raf inhibitors in CRC.

We further demonstrated therapeutic implications of SCNN1B. SCNN1B overexpression was shown to confer increased sensitivity to 5-Fluorouracil, a thymidylate synthase inhibitor and a commonly used chemotherapy in CRC [22]. This implies that SCNN1B might have potential impact on CRC patient survival outcomes. Corroborating these experimental observations, SCNN1B was consistently down-regulated in multiple CRC patient cohorts. Moreover, SCNN1B protein was an independent prognostic factor associated with favorable survival in CRC patients. On the contrary, SCNN1B promoter hypermethylation independently predicts poor survival in CRC, consistent with its role in driving transcriptional silence of SCNN1B. Collectively, SCNN1B represents an actionable target and prognostic factor in CRC.

In summary, we identified for the first time that SCNN1B acts as a tumor suppressor in CRC by targeting of MAPK oncogenic signaling through inactivation of c-Raf, thus impairing MEK-ERK signaling cascades. This in turn is manifested in cell cycle arrest and apoptosis induction, subsequently leading to tumor suppression. In terms of translational relevance, the expression of SCNN1B could serve to stratify CRC patients according to their potential outcomes.

## Materials and Methods

### Cell culture

In this study, ten CRC cell lines were used in this study. CACO-2, COLO205, DLD1, HCT116, HT29, RKO, SW48, SW480, SW620, and SW1116 cells were all obtained from the American Type Culture Collection (ATCC). All cell lines were authenticated by ATCC. Cell lines were cultured in DMEM supplemented with 10% fetal bovine serum and antibiotic-antimycotic, in a humidified cell culture incubator at 37 ^o^C and 5% CO_2_.

### SCNN1B overexpression in CRC cells

Full-length open reading frame of SCNN1B was cloned into pcDNA3.1 [4]; and transfected into DLD1 or SW1116 cells with Lipofectamine 2000 following the manufacturer’s protocol. To obtain stable cell lines overexpressing SCNN1B, cells were selected with G418 for at least 2 weeks. SCNN1B overexpression was validated by qPCR and western blot. Vectors with wildtype c-Raf, c-Raf S29A, and c-Raf S259A were purchased from Addgene. Transient transfection was performed for c-Raf proteins using Lipofectamine 2000.

### Cell viability and colony formation assays

For MTT cell viability assay, cells were seeded onto 96 cell plates. At specified intervals, MTT assay was performed by the addition of 3-(4,5-dimethylthiazol-2-yl)-2,5-diphenyltetrazolium bromide for 4 h, followed by the addition of 0.1 N HCl in 10% SDS. After overnight incubation, plates were read at 570 and 690 nm, respectively. For colony formation assay, cells were seeded into 24-well plates at 200–500 cells per well. After 7–10 days, the cells were fixed and stained with 0.1% crystal violet. The number of cell colonies were then counted.

### Colony formation and cell growth curve

To perform colony formation, cells were plated in 24-well plates at 200 cells/well in complete DMEM. Medium was changed every 3 to 4 days for 10–14 days. At the endpoint, cells were stained with 0.1% crystal violet and the number of colonies (>50 cells) were counted. The cell growth curve was performed by seeding 2000 cells/well in 96-well plates. At each time point, cell viability was determined by 3-(4,5-dimethylthiazol-2-yl)-2,5-diphenyltetrazolium bromide (MTT) assay (Sigma-Aldrich).

### Apoptosis and cell cycle

Cell apoptosis was performed using Annexin V/7-aminoactinomycin D (7-AAD) staining kit (BD Biosciences). For cell cycle, cells were fixed in 70% ethanol and stained with propidium iodide/RNase A (BD Biosciences).

### Cell migration

Cell migration was determined by wound healing assay. Confluent cultures in 6-well plates were scratched with sterile P-200 pipette tips, washed, and cultured in DMEM with 1% FBS. Cells were photographed under a light microscope to determine wound closure rate (%).

### Clinical cohorts

The tissue microarray (TMA) was established using formalin-fixed, paraffin-embedded tissues from 156 patients with CRC that were collected at the Prince of Wales Hospital, Hong Kong from 2002 to 2010, with a median follow-up time of 65.9 months [5, 6]. Paired adjacent normal and CRC tissues were obtained from patients at the Prince of Wales Hospital in Hong Kong to obtain RNA, protein, and paraffin-embedded tissues. All subjects provided informed consent for obtaining the study samples. TMA was stained with anti-SCNN1B antibody (HPA015612; Sigma-Aldrich) and was scored by experienced pathologists. TCGACOAD READ cohort was downloaded using the UCSC Xena Browser (https://xenabrowser.net/). All subjects provided informed consent for the study specimens, and the study was approved the Clinical Research Ethics Committee of the Chinese University of Hong Kong (Hong Kong, China).

### Antibody and PCR Array analysis

MAPK signaling PCR array (Qiagen) was used to determine the effect of SCNN1B on gene expression. Phosphorylation status of MAPK proteins was investigated using human phospho-MAPK antibody array (R&D Systems).

### Luciferase Reporter Assays

Cells were seeded in 24-well plates at 1 × 10^5^ cells/well. After overnight incubation, cells were transfected with serum response element (SRE) luciferase construct, which was co-transfected with Renilla luciferase using Lipofectamine 2000. At 24 h post-transfection, Dual-Luciferase Reporter Assay System (Bio-Rad, CA) was employed to detect Firefly and Renilla luciferase activities. All the Firefly luciferase data were normalized to Renilla luciferase.

### Subcutaneous xenograft model

DLD1 (2 × 10^6^ cells in 0.1 mL PBS) and SW1116 (1 × 10^7^ cells in 0.1 mL PBS) cells stably expressing empty vector or SCNN1B were injected subcutaneously into the left and right dorsal flank of 6-week-old female BALB/c nude mice (*n* = 6/group), respectively. Tumor size was measured twice a week using a digital caliper. Tumor volume (*V*) was estimated by measuring the longest diameter (L) and shortest diameter (W) of the tumor. Tumor volume was calculated using the below formula *V* = 0.5 x L x W^2^. Sample size was determined according to previous studies [4], and no randomization or blinding was performed. No animals were excluded from analysis. All experimental procedures were approved by the Animal Ethics Committee of the Chinese University of Hong Kong (Hong Kong, China).

### KRAS and c-Raf activity assays

Active Ras Pull-Down and Detection Kit (ThermoFisher) was used to determine active KRAS status in cells. Pulled-down active KRAS was detected using anti-KRAS antibody (ab180772). For c-Raf kinase activity assay was obtained from BPS bioscience, and performed according to the manufacturer’s protocol.

### Statistical analysis

Data are presented as mean ± standard error (S.E.M.) or described as frequency and percentage. In vitro studies were performed in at least triplicates in 2 independent experiments. To compare the difference between 2 groups, two-tailed student’s *t*-test or Mann-Whitney U test was used. Variance was similar between groups that were statistically compared. The difference between growth curves was determined by two-way ANOVA. Kaplan-Meier analysis and log-rank test were performed to evaluate patient survival in relation to SCNN1B expression. Hazard ratios was estimated with the univariate and multivariate Cox regression models using SPSS software (IBM). Other statistical analyses were performed using GraphPad Prism, Version 6.0. *P* < 0.05 was considered statistically significant.

## Supplementary information

Supplemental Figures

Supplemental Tables

## Data Availability

All primary data is available from the corresponding author upon request.
