# Supplementary material for: The sodium channel subunit SCNN1B suppresses colorectal cancer via suppression of active c-Raf and MAPK signaling cascade
Source: Oncogene. 2022 Dec 23;42(8):601–12. doi: 10.1038/s41388-022-02576-4 (PMC9937924; doi:10.1038/s41388-022-02576-4)
Supplement: Supplementary file 1 — Supplemental Figures [file 41388_2022_2576_MOESM1_ESM.pdf]

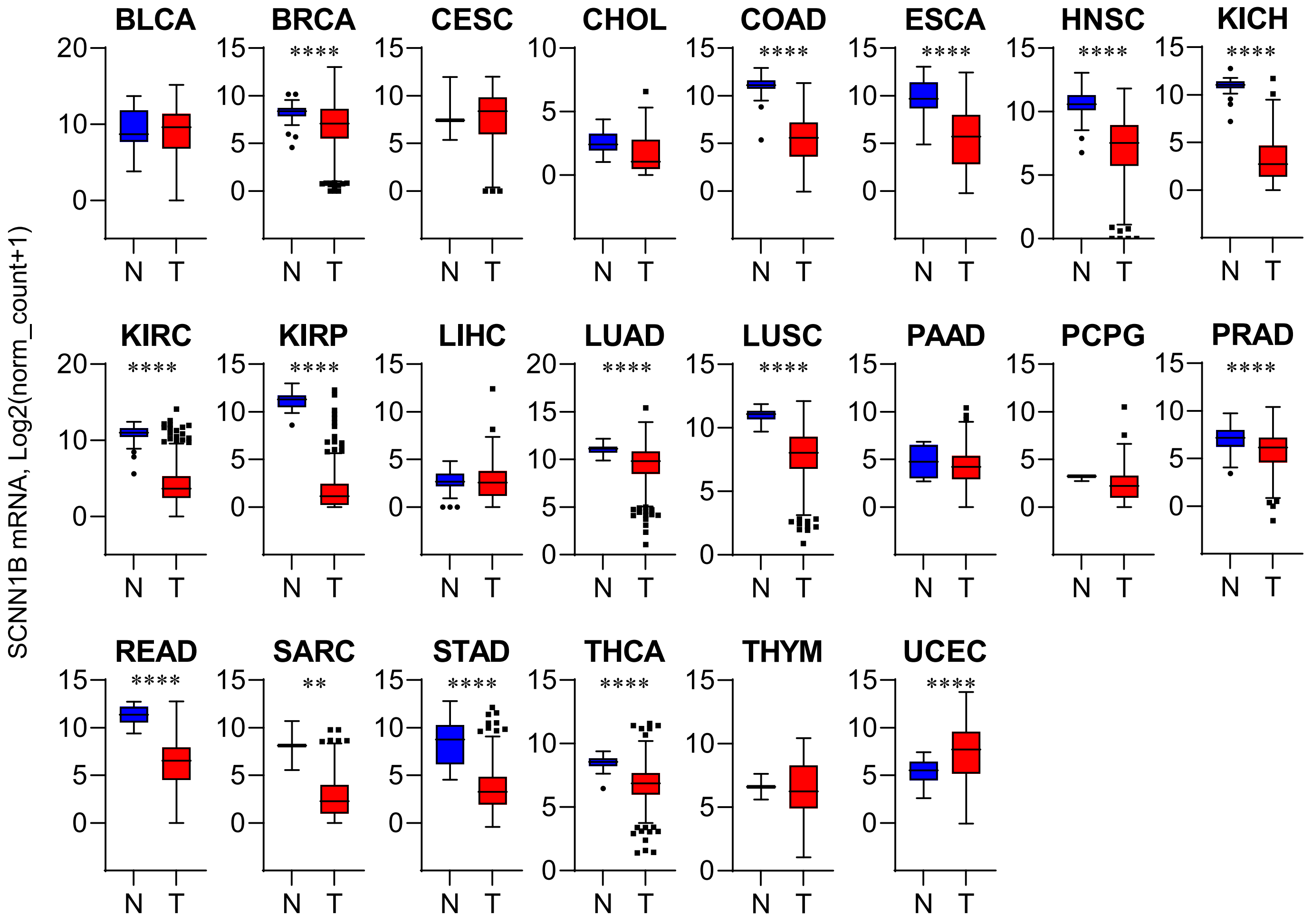

Figure S1. SCNN1B mRNA expression in TCGA database. SCNN1B mRNA expression is silenced in a number of cancer types in TCGA cohort.

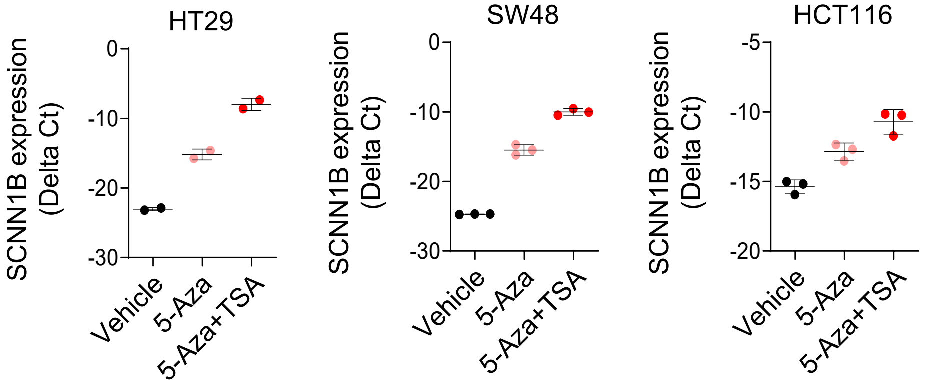

Figure S2. Mechanism of SCNN1B silence in CRC cells. SCNN1B mRNA was restored in CRC cells treated with the combination of 5-Aza and trichostatin A (TSA), a histone deacetylase inhibitor.

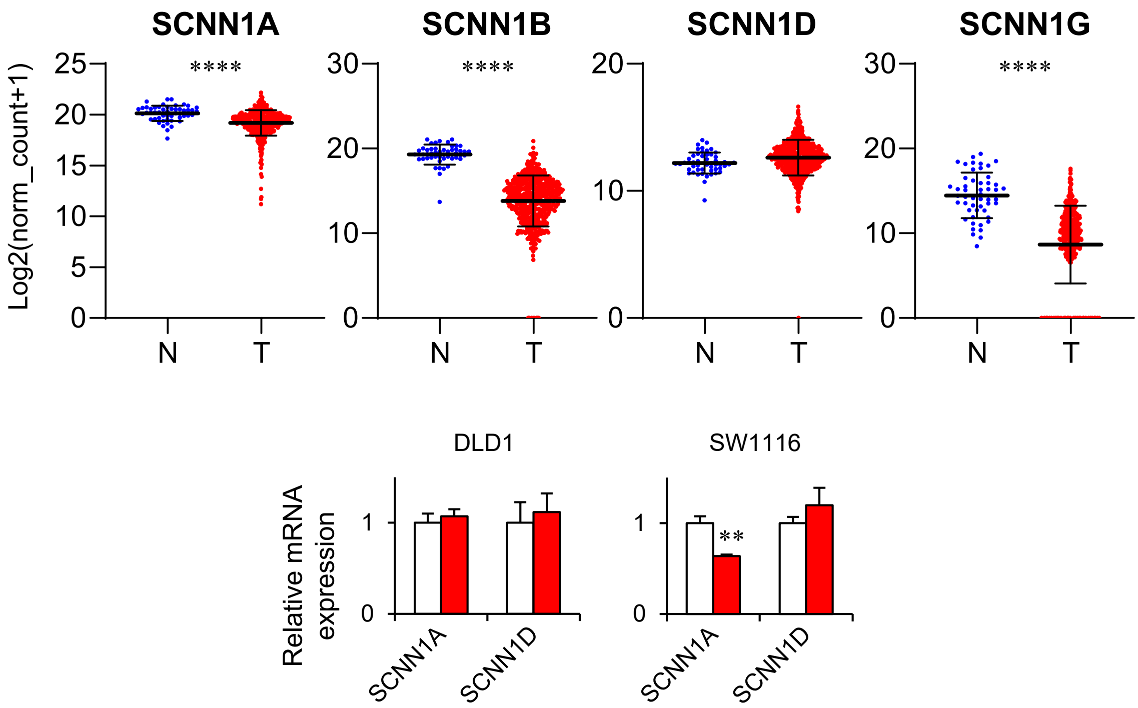

Figure S3. SCNN1B had no effect on other ENaC subunits. SCNN1A and SCNN1G are down-regulated in TCGA CRC cohort (upper). SCNN1B overexpression had no consistent effect on other ENaC subunits (lower).

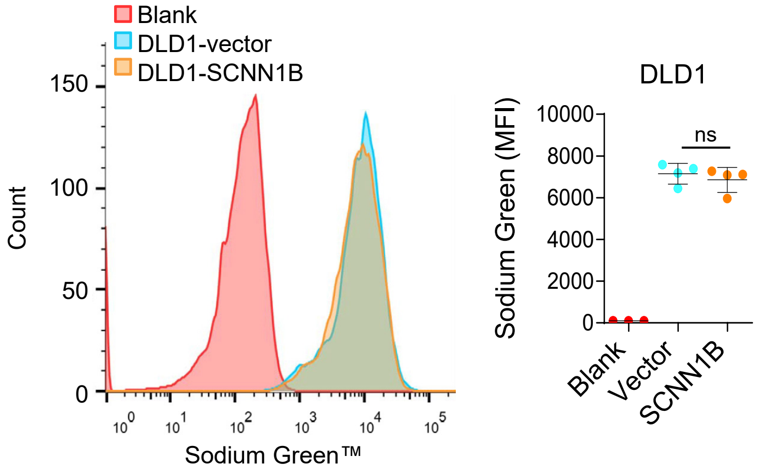

Figure S4. SCNN1B had no effect on sodium content of CRC cells. Flow cytometry of cellular sodium content after staining of DLD1-vector and DLD1-SCNN1B cells wit Sodium Green^{TM} dye for 30min at room temperature.

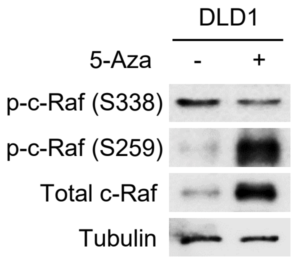

Figure S5. Effect of 5-Aza on c-Raf status. 5-Aza treatment in DLD1 cells promoted c-Raf S259 phosphorylation while suppressing S338 phosphorylation.

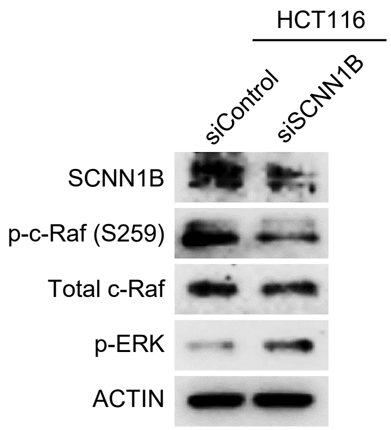

Figure S6. Effect of SCNN1B knockdown on c-Raf activation. SCNN1B knockdown in HCT116 cells suppressed S259 phosphorylation, but activated p-ERK.

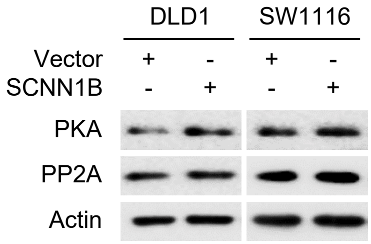

Figure S7. SCNN1B had no effect on c-Raf upstream regulators. Expression of protein kinase A (PKA) and protein phosphatase 2A (PP2A) was determined in SCNN1B-overexpressing cell lines by western blot.

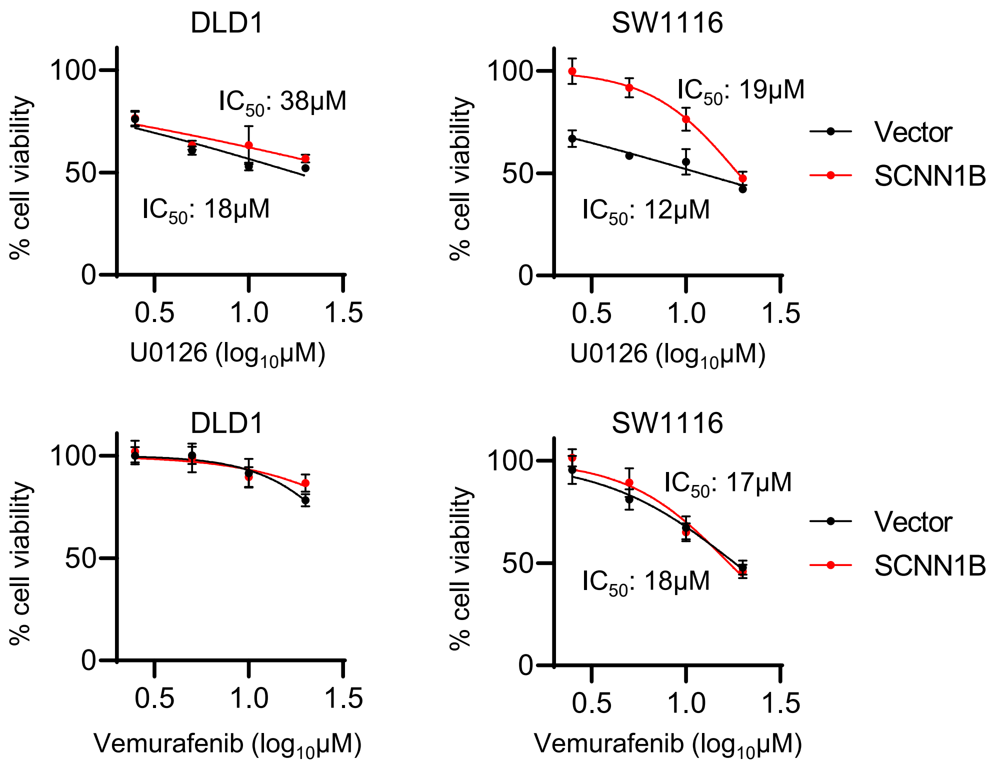

Figure S8. Effect of SCNN1B overexpression on antiproliferative effect (72h-IC_{50}) of U0126 (ERK inhibitor) and Vemurafenib (BRAF inhibitor).
