# Supplementary material for: The sodium channel subunit SCNN1B suppresses colorectal cancer via suppression of active c-Raf and MAPK signaling cascade
Source: Oncogene. 2022 Dec 23;42(8):601–12. doi: 10.1038/s41388-022-02576-4 (PMC9937924; doi:10.1038/s41388-022-02576-4)
Supplement: Supplementary file 2 — Supplemental Tables [file 41388_2022_2576_MOESM2_ESM.docx]

**Table S1.** Primer Sequences Used in This Study

| **Primer Name** | **Sequence (5’-3’)** | **Application** |
| --- | --- | --- |
| SCNN1B-F | AGTGCTACCCAGGCATTGAC | RT-PCR |
| SCNN1B-R | GTCATGCCCCAGTTGAAGAT |  |
| SCNN1B-F | AGACAACCACAATGGCTTAACA | Real time-PCR |
| SCNN1B-R | TGAGGCTACATAGTCTCATGGC |  |
| SCNN1B-BGS-F | GTTTAGTGTTTTTGAATTTGG | BGS |
| SCNN1B-BGS-R | CTTCTACACCCTAAAAACTTTTCC |  |
| SCNN1B-MSP-M-F | GTGTGGTTAGGTCGGTAGC | MSP |
| SCNN1B-MSP-M-R | AACACTAAAACACCCGACG |  |
| SCNN1B-MSP-U-F | GTGTGGTTAGGTTGGTAGT | MSP |
| SCNN1B-MSP-U-R | AACACTAAAACACCCAACA |  |
| Actin-F | AGAGCTACGAGCTGCCTGAC | RT-PCR & Real time-PCR |
| Actin-R | AGCACTGTGTTGGCGTACAG |  |

**Table S2.** Antibodies Used in This Study

| **Protein** | **Cat #** | **Company** | **Application** |
| --- | --- | --- | --- |
| SCNN1B | HPA015612 | Sigma-Aldrich | WB/IHC |
| GAPDH | sc-25778 | Santa Cruz | WB |
| β-Actin | sc-47778 | Santa Cruz | WB |
| Caspase-8 | 9746 | Cell Signaling | WB |
| Caspase-9 | 9508 | Cell Signaling | WB |
| Cleaved caspase-7 | 9492 | Cell Signaling | WB |
| Caspase-7 | 9491 | Cell Signaling | WB |
| Cleaved PARP | 5625 | Cell Signaling | WB |
| PARP | 9532 | Cell Signaling | WB |
| p21 | sc-6246 | Santa Cruz | WB |
| p27^kip1^ | 3686 | Cell Signaling | WB |
| p53 | sc-126 | Santa Cruz | WB |
| MMP-9 | 13667 | Cell Signaling | WB |
| Cyclin D1 | 2978 | Cell Signaling | WB |
| p-AKT1 (Ser473) | 9018 | Cell Signaling | WB |
| p-AKT2 (Ser474) | 8599 | Cell Signaling | WB |
| p-MEK1/2 (Ser217/221) | 9154 | Cell Signaling | WB |
| p-ERK1/2 (Thr202/Tyr204) | 4370 | Cell Signaling | WB |
| p-A-Raf (Ser299) | 4431 | Cell Signaling | WB |
| A-Raf | 4432 | Cell Signaling | WB |
| p-B-Raf (Ser445) | 2696 | Cell Signaling | WB |
| B-Raf (55C6) | 9433 | Cell Signaling | WB |
| p-c-Raf (Ser338) | 9427 | Cell Signaling | WB |
| p-c-Raf (Ser289/296/301) | 9431 | Cell Signaling | WB |
| p-c-Raf (Ser259) | 9421 | Cell Signaling | WB |
| c-Raf (D5X6R) | 12552 | Cell Signaling | WB |
| Anti-cAMP Protein Kinase | ab76238 | Abcam | WB |
| Anti-PP2A | ab32104 | Abcam | WB |
| Anti-Flag | F1804 | Sigma-Aldrich | WB |
